# Modelling food allergy with mice models

**DOI:** 10.1186/2045-7022-1-S1-S44

**Published:** 2011-08-12

**Authors:** Udo Herz

**Affiliations:** 1 Mead Johnson Nutrition, Research & Development, Nijmegen, Netherlands

## 

Food allergies are adverse immune reactions to food proteins that can range from immediate, potentially life-threatening reactions to chronic disorders such as atopic dermatitis and allergic gastrointestinal disorders. These adverse reactions can be IgE-mediated; cells mediated or result from a combination of both. No effective preventive strategy or curative protocol is currently established. Various mouse models have been developed which mirror some of the key elements of food allergies to a high degree. These models have extended our understanding on the immunological and patho-physiological mechanisms of the allergic immune response and have been used for the initial testing of preventive and therapeutic agents. In particular the prenatal and early postnatal period seem to be a critical window for the establishment and maintenance of a normal immune response towards food allergens. This presentation will focus on the role of the maternal adaptive immune response and the nature of the diaplacental antigen transfer during the prenatal period in preventing the onset of allergies in the offspring. In addition, during the early postnatal period several host factors can influence the acquisition of oral tolerance. The interaction of the developing immune system with microbial structures seems to play a decisive role for the induction of local and systemic tolerance. Several studies demonstrated that continuous administration of live Lactobacillus rhamnosus GG (LGG) during gestation and the breastfeeding period inhibited the onset of allergen-induced sensitization and airway disease in the offspring which is associated with the induction of T-regulatory cells. Recent findings suggest that heat treated and soluble factors may also have the ability to suppress the allergic immune response. These data may help to interpret previous data from successful clinical trials and provide an outlook on future intervention strategies.

